# The relationship between AI anxiety and academic motivation among university students: the mediating role of emotion regulation and the moderating role of gender

**DOI:** 10.3389/fpsyg.2026.1918525

**Published:** 2026-09-04

**Authors:** Yuanchang Zhang, Yantao Shi, Jing Lu

**Affiliations:** 1 School of Modern Agriculture and Food Engineering, Chuzhou University, Chuzhou, Anhui, China; 2 College of Innovation and Entrepreneurship, Guangxi Minzu University, Nanning, China; 3 Rural Industry Revitalization School of Wuzhou Vocational College, Wuzhou, China

**Keywords:** academic motivation, AI anxiety, emotion regulation, gender, moderated mediation model, university students

## Abstract

**Background:**

Generative artificial intelligence (AI) is increasingly embedded in university students’ writing, information retrieval, knowledge organisation and task completion. Although AI tools may improve learning convenience and access to resources, they may also generate technological uncertainty, pressure to adapt to new competencies and concerns about future development. As an important negative emotional response in intelligent technology contexts, AI anxiety may be closely associated with university students’ academic motivation and psychological adaptation.

**Methods:**

This cross-sectional questionnaire study recruited 1,484 university students in China using convenience sampling. Participants completed measures of AI anxiety, emotion regulation and academic motivation through the Wenjuanxing online survey platform. Descriptive statistics, Pearson correlation analysis and regression analyses were used to examine associations among variables. The PROCESS macro was used to test the mediation effect and the moderated mediation effect. All indirect effects were estimated using 5,000 bootstrap samples and 95% confidence intervals.

**Results:**

AI anxiety was negatively associated with both emotion regulation and academic motivation, whereas emotion regulation was positively associated with academic motivation. Bootstrap analyses showed a significant negative indirect association between AI anxiety and academic motivation through emotion regulation. Gender significantly moderated the association between emotion regulation and academic motivation, with a stronger positive association among male students.

**Conclusion:**

AI anxiety may constitute a psychological barrier to students’ motivational adaptation to AI-supported learning. Emotion regulation represents one psychological pathway linking AI anxiety to academic motivation, and gender constitutes a boundary condition for this association. These findings suggest that AI literacy education should integrate emotion-regulation and metacognitive support with differentiated learning assistance. Given the cross-sectional and self-reported nature of the data, the findings require further validation using longitudinal or experimental designs.

## 1 Background

Artificial intelligence, particularly generative AI, is profoundly changing learning practices in higher education. In China, the rapid development of large language models and the increasing integration of AI tools into university teaching and learning have accelerated the transformation of educational practices. Universities have begun exploring AI-supported teaching, intelligent learning platforms and AI literacy education, while students increasingly use generative AI for writing assistance, information retrieval, knowledge organisation and learning decisions. In Chinese higher education, generative AI has been integrated into classroom teaching, independent learning, academic writing and research support. However, differences in institutional guidance, teacher preparedness and students’ AI literacy may create adaptation challenges. When university students use AI tools for writing assistance, information retrieval, knowledge organisation and learning decisions, they may benefit from increased efficiency and resource support. At the same time, they may face algorithmic opacity, blurred boundaries of academic integrity, technological dependence, perceived competence replacement and uncertainty about future careers (Chan and Hu, 2023; Crompton and Burke, 2023; Hu, 2026; Kim et al., 2025). Empirical studies have shown that while generative AI improves learning efficiency, feedback convenience and flexible access to knowledge, it also introduces new challenges for academic integrity, ethical judgement and the boundaries of learning responsibility in higher education (Bittle and El-Gayar, 2025; Kasneci et al., 2023; Kofinas et al., 2025; Lund et al., 2025; O’Dea, 2024). As AI becomes increasingly embedded in students’ daily learning environments, psychological responses toward AI have become an important issue in higher education. Students’ concerns about AI complexity, uncertainty, and potential threats to academic competence and future employability are increasingly reported, suggesting that AI-related emotional experiences may influence how students adapt to AI-supported learning environments. The resulting worry, tension and reduced sense of control constitute important manifestations of AI anxiety. Although national prevalence data remain limited, AI-related worry, uncertainty and reduced perceived control are increasingly reported among university students. These experiences are important because they may affect students’ adaptation and motivation in AI-supported learning. In this study, AI anxiety refers to a situation-specific emotional response arising from both students’ uncertainty about how to use AI appropriately and their concerns about the potential academic, ethical and career-related consequences of AI use. Unlike a general negative attitude toward technology, AI anxiety emphasises emotional arousal and psychological pressure during contact with, understanding of and use of AI technology, reflecting students’ subjective perceptions of AI complexity, uncontrollability and potential consequences (Alshaibani et al., 2025; Budhathoki et al., 2024; Uludağ et al., 2025; Zhao et al., 2025).

Academic motivation refers to students’ subjective judgements about the value of learning activities, their likelihood of success and their own capabilities. It is directly related to goal orientation, effort and sustained engagement. Both self-determination theory and expectancy-value theory suggest that students’ willingness to engage in learning depends not only on external requirements but also on their evaluations of learning meaning, competence development and behavioural control (Eccles and Wigfield, 2020; Guay, 2022; Litalien et al., 2024; Schweder and Raufelder, 2022). Meta-analytic evidence indicates that autonomous motivation, controlled motivation and amotivation are differentially associated with academic performance, persistence, well-being and academic adjustment, and that the quality of motivation explains differences in learning outcomes more effectively than motivation intensity alone (Bureau et al., 2022; Howard et al., 2021). When AI enters higher education learning contexts, students need to reconsider how knowledge is acquired, how learning responsibility is distributed and how their own abilities can be developed. Generative AI not only changes the provision of learning resources but may also reshape students’ judgements of task value, competence improvement and future competitiveness (Al-Mamary and Abubakar, 2025; Chen and Cheung, 2025; Hmoud et al., 2024; Kim et al., 2025; Moorhouse et al., 2025). However, existing research has mainly focused on the educational affordances and ethical challenges of generative AI, while less attention has been paid to how students’ psychological responses to AI-related uncertainty may influence their academic motivation. Therefore, AI anxiety may be associated with academic motivation by influencing students’ perceptions of technological adaptation, competence development and the necessity of continued learning.

Previous research has focused mainly on the relationships between AI anxiety and AI adoption intention, technology acceptance and AI learning behaviour, whereas less attention has been paid to the psychological processes and boundary conditions through which AI anxiety is linked to university students’ academic motivation. Existing studies suggest that AI anxiety affects students’ willingness to adopt AI tools, intentions to use ChatGPT and AI learning behaviour, and that these associations are shaped by factors such as self-efficacy, perceived value, social influence and technological trust (Arthur et al., 2024; Cao et al., 2026; Chen J. et al., 2025; Strzelecki, 2024; Strzelecki and ElArabawy, 2024; Uludağ et al., 2025; Wang et al., 2024). Recent studies on AI-assisted writing and academic integrity further indicate that students do not have a consistent understanding of AI use norms, ethical boundaries and the consequences of misuse, and that this normative uncertainty may intensify psychological pressure and motivational fluctuations during learning (Bittle and El-Gayar, 2025; Gonsalves, 2025; Lund et al., 2025). As AI-supported learning becomes increasingly normalised, students must adapt to both technological convenience and technological pressure. AI anxiety may undermine learning confidence and motivational persistence by increasing emotional strain and reducing perceived control over learning. It is therefore necessary to examine the mechanisms linking AI anxiety and academic motivation.

### 1.1 Theoretical framework

#### 1.1.1 AI anxiety and academic motivation among university students

This study uses self-regulated learning theory to explain the relationship between AI anxiety and academic motivation among university students. MASRL provides a more detailed account of metacognitive and affective processes at the task level, but the present study examines a broader regulatory pathway from AI-related emotion to academic motivation. Therefore, MASRL is treated as a complementary perspective rather than the primary framework. Although MASRL (Efklides, 2011) highlights learners’ emotional processes, SRL was adopted because it provides a broader framework integrating cognition, emotion, motivation and behaviour, which is suitable for explaining how AI-related emotional experiences are associated with learning motivation. This theory posits that students actively regulate cognition, motivation and behaviour in response to learning tasks and environmental changes (Boekaerts, 1999; Zimmerman, 2000). In this study, the learning context refers not to the university environment in general but to specific AI-supported learning tasks, such as academic writing, information retrieval, content evaluation and task completion, in which students set goals, monitor AI-generated outputs and adjust their learning strategies. Although SRL was developed before generative AI, The core regulatory processes proposed by SRL are not technology-specific; rather, they explain how learners adapt their goals, emotions and strategies to changing learning conditions. Although SRL predates generative AI, its emphasis on the adaptive regulation of cognition, emotion and behaviour remains relevant to AI-supported learning. Generative AI introduces a new learning condition that requires students to evaluate the reliability of AI-generated content, adjust their learning strategies and reconsider their roles in knowledge acquisition (Pallant et al., 2025; Silvola et al., 2025; Uludağ et al., 2025). At the same time, AI-related uncertainty, competence requirements and future development pressures may consume regulatory resources and weaken students’ perceived control, thereby constraining academic motivation (Cao et al., 2026; Galindo-Domínguez et al., 2026; Wang et al., 2024). From the SRL perspective, AI anxiety refers to students’ emotional uncertainty and concerns about generative AI use; emotion regulation refers to their ability to manage AI-related emotional responses; and academic motivation reflects their willingness, value perception and goal orientation toward learning.

AI-related uncertainty may consume the cognitive and emotional resources required for learning regulation. Concerns about occupational displacement, technological reliability, and changing competence requirements may reduce students’ perceived control and confidence in meeting academic demands (Cao et al., 2026; Teke, 2026; Wang et al., 2024; Zhang et al., 2026). Similarly, AI self-efficacy, social influence, and technological trust shape how students respond to AI-supported learning (Chen et al., 2025; Uludağ et al., 2025). Higher AI anxiety is therefore expected to be associated with lower academic motivation.

### 1.2 Mediating role of emotion regulation

Emotion regulation refers to the processes through which individuals identify, monitor, and modify their emotional responses, helping students maintain goal orientation and behavioural control under demanding learning conditions (Gross, 2015). Self-regulated learning theory emphasises the coordination of cognitive, emotional, motivational, and behavioural regulation (Boekaerts, 1999; Zimmerman, 2000). Within this framework, emotional states influence students’ metacognitive monitoring, strategy selection, and allocation of attentional resources. Anxiety may disrupt this regulatory process by directing attention toward perceived threats and weakening perceived control over learning. In AI-supported learning, students must evaluate the accuracy, applicability, and academic boundaries of AI-generated content, which may increase technological uncertainty and psychological strain (Chan and Hu, 2023; Crompton and Burke, 2023; Luo et al., 2026; Tao et al., 2026).

AI anxiety may be negatively associated with emotion regulation. From a self-regulated learning perspective, AI anxiety is an affective condition that may interfere with students’ monitoring and regulation of AI-supported learning. Concerns about technological complexity, competence displacement, academic integrity, and sociotechnical uncertainty may consume attentional and emotional resources (Cao et al., 2026; Wang et al., 2024). Persistent worry and reduced perceived control may, in turn, make it more difficult for students to modify negative emotions. Consequently, students may have fewer regulatory resources available to reappraise negative experiences, maintain learning goals, and adapt their learning strategies. Although adaptive emotion-regulation strategies support academic adjustment, heightened anxiety may constrain their effective use (Iuga and David, 2024; Marques et al., 2023; Romo et al., 2025; Wang et al., 2025). Therefore, students with higher AI anxiety are expected to report lower levels of emotion regulation.

Emotion regulation may serve as an important personal resource associated with university students’ academic motivation. According to study demands–resources theory, study demands can deplete psychological resources, whereas personal resources help students cope with pressure, maintain goal orientation, and sustain their willingness to learn (Bakker, 2024; Bakker and Demerouti, 2017; Garn and Stenling, 2024; Zeijen et al., 2024). Emotion regulation can therefore be regarded as a personal resource that enables students to manage negative emotional responses and maintain motivational functioning under stressful and uncertain conditions (Gross, 2015; Iuga and David, 2024).

Students with stronger emotion-regulation capacities may be better able to manage AI-related uncertainty, reduce emotional interference, and preserve perceived control over learning. By contrast, difficulties in emotion regulation may allow anxiety and frustration to disrupt goal persistence and willingness to learn. Previous research has similarly linked adaptive emotion regulation to stronger academic adjustment, lower academic burnout, and better academic functioning (Iuga and David, 2024; Kryshko et al., 2023; Romo et al., 2025; Schwinger et al., 2022). Emotion regulation is therefore expected to be positively associated with academic motivation in AI-supported learning contexts.

Taken together, AI anxiety may be negatively associated with academic motivation through reduced emotion regulation. Students experiencing greater AI anxiety may find it more difficult to manage emotional responses to technological uncertainty and perceived competence threats. Lower emotion regulation may, in turn, be associated with reduced goal persistence, perceived learning control, and willingness to sustain academic effort. Emotion regulation may therefore constitute an explanatory psychological pathway linking AI anxiety to academic motivation.

### 1.3 Moderating role of gender

Previous research has shown that emotion regulation is an important psychological mechanism influencing university students’ academic motivation (Fong et al., 2024; Liang and Mao, 2025; Rentzios et al., 2025). Emotion regulation theory suggests that when individuals encounter stress, frustration or negative emotional experiences, they may manage emotional responses through situation selection, attentional deployment, cognitive change and response modulation (Gross, 1998, 2015). A meta-analysis has shown that emotion regulation strategies can effectively influence emotional experience, behavioural responses and physiological outcomes (Webb et al., 2012). In learning processes, effective emotion regulation helps reduce the interference of negative emotions with cognitive processing, goal persistence and motivational engagement, thereby maintaining academic motivation. In contrast, insufficient emotion regulation may increase vulnerability to academic frustration, learning avoidance and motivational decline (Aldao et al., 2010; Pekrun et al., 2002; Pekrun, 2006). University students need to regulate not only cognition and behaviour during learning but also emotional responses triggered by learning tasks, evaluative pressure and blocked goals (Ben-Eliyahu and Linnenbrink-Garcia, 2013; Harley et al., 2019). Previous research has shown that emotions, self-regulated learning and academic motivation jointly influence academic performance (Mega et al., 2014). Thus, emotion regulation can be regarded as a learning-related psychological resource that helps university students manage emotional responses and maintain academic motivation.

The association between emotion regulation and academic motivation may differ by gender. Previous studies have found differences in the use of emotion regulation strategies such as cognitive reappraisal and expressive suppression, and these differences are closely associated with emotional experience, social functioning and mental health (Gross and John, 2003). Meta-analytic evidence suggests that women are more likely to use emotional expression, reflection and emotional support seeking, whereas men tend to rely relatively more on emotional control, problem-focused coping or self-regulation when facing stress (Tamres et al., 2002). Other research also indicates that men and women may show different neural activation patterns when using cognitive reappraisal to regulate negative emotions (McRae et al., 2008). AI-specific evidence also suggests possible gender differences in AI-related anxiety. Recent studies of nursing, midwifery and dental students found that female students reported higher overall AI anxiety than male students, including greater concerns about AI configuration and sociotechnical uncertainty (Güner and Aydın, 2026; Karaaslan and Erişken, 2026). However, these findings appear to depend on academic discipline, cultural context and technological experience. Moreover, gender differences in mean AI-anxiety levels do not directly demonstrate that gender moderates the association between emotion regulation and academic motivation; rather, they support considering gender as a potential boundary condition in students’ psychological adaptation to AI.

Based on this evidence, the strength of the association between emotion regulation and academic motivation may differ between male and female university students. Male students may be relatively more likely to rely on internal regulation and self-control when coping with academic setbacks; therefore, stronger emotion regulation may be more closely associated with their ability to manage negative emotions, maintain goal-directed behaviour, and sustain academic motivation. By contrast, female students’ academic motivation may additionally draw on emotional expression, interpersonal communication, and social support. Accordingly, although emotion regulation is expected to be positively associated with academic motivation in both groups, this association may be stronger among male students.

### 1.4 The present study

Based on the preceding theoretical reasoning, this study constructed a moderated mediation model to examine the association between AI anxiety and academic motivation, the mediating role of emotion regulation, and the moderating role of gender. Accordingly, we proposed the following hypotheses:

*H1:* We hypothesized that AI anxiety would be negatively associated with academic motivation among university students.

*H2a:* We hypothesized that AI anxiety would be negatively associated with emotion regulation among university students.

*H2b:* We hypothesized that emotion regulation would be positively associated with academic motivation among university students.

*H2:* We hypothesized that AI anxiety would have a negative indirect association with academic motivation through emotion regulation.

*H3:* We hypothesized that gender would moderate the positive association between emotion regulation and academic motivation, such that this association would be stronger among male than female university students.

The proposed model was intended to clarify whether AI anxiety was indirectly associated with academic motivation through reduced emotion regulation and whether the second stage of this indirect association differed by gender. The findings may contribute to understanding university students’ psychological adaptation to AI-supported learning and inform AI literacy education, learning support, and differentiated psychological interventions in higher education.

## 2 Methods

### 2.1 Participants

A total of 1,484 Chinese college students were included in this study. Participants were recruited using convenience sampling and completed an online questionnaire through Questionnaire Star (www.wjx.cn). Of the participants, 823 were female (55.5%) and 661 were male (44.5%). Regarding daily AI use, 1,077 participants (72.6%) used AI for less than 1 h per day, 278 (18.7%) for 1–2 h, 56 (3.8%) for 2–3 h, and 73 (4.9%) for more than 3 h. Daily AI use time was included as a control variable in the subsequent analyses.

### 2.2 Measures

#### 2.2.1 Emotion regulation

Emotion regulation was measured with the Emotion Regulation Questionnaire (Gross and John, 2003). The 10-item scale includes cognitive reappraisal and expressive suppression. Items were rated from 1 (strongly disagree) to 6 (strongly agree). Higher average scores indicate greater overall use of emotion regulation strategies; therefore, the total score should be interpreted as general strategy use rather than as cognitive reappraisal alone. Because the items were measured using ordered Likert-type response categories, the two-factor CFA was estimated using the WLSMV estimator. Model fit was evaluated using χ^2^, df, χ^2^/df, CFI, TLI, RMSEA, and SRMR. The two-factor CFA showed acceptable fit, χ^2^ = 45.399, df = 34, χ^2^/df = 1.335, CFI = 0.998, TLI = 0.997, RMSEA = 0.015, and SRMR = 0.046. Cronbach’s alpha was 0.896 for the overall scale.

#### 2.2.2 Academic motivation

Academic motivation was assessed with the Academic Motivation Scale based on the Revised Study Process Questionnaire (Biggs et al., 2001). The 20-item scale includes deep and surface motivation. Items were rated from 1 (completely inconsistent) to 5 (completely consistent). Mean scores were used in the main analyses to indicate overall academic motivation, while the two-dimensional structure was retained in the measurement evaluation. Consistent with the other measurement models, the second-order CFA was estimated using the WLSMV estimator, and the same set of fit indices was reported. The second-order CFA supported the hierarchical model, χ^2^ = 1363.099, df = 169, χ^2^/df = 8.066, RMSEA = 0.069, TLI = 0.976, and CFI = 0.979. SRMR = 0.083. Cronbach’s alpha was 0.934 for the total scale.

#### 2.2.3 AI anxiety

AI anxiety was measured with four items adapted from the technology-anxiety construct in the Unified Theory of Acceptance and Use of Technology (Venkatesh et al., 2003). The wording was revised to refer to AI systems in learning contexts and reviewed by researchers in educational psychology, educational technology, and scale development. Items were rated from 1 (strongly disagree) to 5 (strongly agree). Higher scores indicate stronger anxiety about AI use. Consistent with the analyses for the other scales, the one-factor CFA was estimated using the WLSMV estimator, and model fit was evaluated using χ^2^, df, χ^2^/df, CFI, TLI, RMSEA, and SRMR. The one-factor CFA showed strong CFI, TLI, and SRMR but elevated RMSEA in a very low-degree-of-freedom model, χ^2^ = 9.301, df = 2, χ^2^/df = 4.651, CFI = 0.998, TLI = 0.993, RMSEA = 0.050, and SRMR = 0.029. Cronbach’s alpha was 0.870.

### 2.3 Procedure

This study was approved by the Research Ethics Committee of the authors’ university. Before accessing the questionnaire items, participants viewed an online informed-consent statement describing the study’s purpose, voluntary nature, anonymity, confidentiality, and right to withdraw. Only participants who provided informed consent proceeded to the questionnaire. Data were collected in September 2025 using Questionnaire Star (www.wjx.cn), a commonly used online survey platform in China. The questionnaire link was distributed to eligible college students, who accessed and completed the survey using their own electronic devices. Participants completed the questionnaire independently and submitted their responses electronically through the platform. The three measurement instruments were presented in a fixed order: (1) the Emotion Regulation Questionnaire, (2) the Academic Motivation Scale, and (3) the AI Anxiety Scale. The order of the instruments was not randomized across participants. Participants were informed that their responses would remain anonymous and confidential and that they could discontinue participation at any time without penalty.

### 2.4 Data analysis

Descriptive statistics and Pearson correlations were first calculated for the study variables (Table 1). The hypothesized indirect association between AI anxiety and academic motivation through emotion regulation was then examined using Model 4 of the PROCESS macro (Hayes, 2013; Table 2). Next, PROCESS Model 14 was used to test whether gender moderated the association between emotion regulation and academic motivation and, consequently, the indirect association between AI anxiety and academic motivation through emotion regulation (Table 3). Conditional indirect effects and the index of moderated mediation were evaluated using bias-corrected 95% confidence intervals based on 5,000 bootstrap resamples. An indirect or moderated mediation effect was considered statistically significant when its bootstrap confidence interval did not include zero.

**Table 1:** Descriptive statistics and correlations among study variables.

Variables	*M*	*SD*	1	2	3	4	5
1. Gender	−	−	1				
2. Daily AI use	2.03	0.923	0.037	1			
3. AI anxiety	3.44	0.801	−0.001	0.029	1		
4. Emotion regulation	4.58	0.894	−0.001	0.007	−0.172**	1	
5. Academic motivation	3.13	0.643	−0.094**	0.053*	−0.175**	0.457**	1

*p < 0.05; **p < 0.01; *** *p* < 0.001.

**Table 2:** Mediation effect of AI anxiety on academic motivation through emotion regulation.

Variables	Model 1 Emotion regulation				Model 2 Academic motivation				Model 3 Academic motivation			
	*β*	SE	LLCI	ULCI	*β*	SE	LLCI	ULCI	*β*	SE	LLCI	ULCI
AI anxiety	−0.172*	0.026	−0.222	−0.122	−0.101*	0.023	−0.147	−0.056	−0.177*	0.026	−0.227	−0.127
Emotion regulation	—	—	—	—	0.439*	0.023	0.393	0.485	—	—	—	—
Daily AI use	0.013	0.028	−0.042	0.067	0.057*	0.025	0.008	0.106	0.063*	0.028	0.008	0.117
R^2^	0.03				0.221				0.034			
F	22.604*				140.080*				26.090*			

*p < 0.05; **p < 0.01; ***p < 0.001. β, standardized coefficient; SE, standard error; LLCI and ULCI indicate the lower and upper limits of the 95% confidence interval, respectively. The indirect effect was tested using 5,000 bootstrap samples.

**Table 3:** Regression results for the moderating effect of gender on the association between emotion regulation and academic motivation.

Variable	*B*	SE	*t*	*p*	LLCI	ULCI
Constant	0.186	0.089	2.102	0.036	0.012	0.359
AI anxiety	−0.104	0.023	−4.521	< 0.001	−0.150	−0.059
Emotion regulation	0.679	0.072	9.427	< 0.001	0.538	0.821
Gender	−0.192	0.046	−4.190	< 0.001	−0.281	−0.102
Emotion regulation × gender	−0.160	0.046	−3.520	< 0.001	−0.250	−0.071
Daily AI use duration	0.055	0.025	2.231	0.026	0.007	0.104

B, unstandardised coefficient; SE, standard error; LLCI and ULCI indicate the lower and upper limits of the 95% confidence interval, respectively. Gender was coded as male = 1 and female = 2. The model controlled for daily AI use duration. Model summary: R^2^ = 0.237, F(5, 1,478) = 91.642, *p* < 0.001.

Preliminary diagnostic analyses were conducted before hypothesis testing (Supplementary Table S1). The continuous variables showed no substantial distributional abnormalities, with absolute skewness values below 1 and kurtosis values below 3. Although the Shapiro–Wilk tests were statistically significant, these results were interpreted cautiously given the sensitivity of significance-based normality tests in large samples. The residual distributions also exhibited acceptable skewness and kurtosis. After centering the variables involved in the interaction term, VIF values ranged from 1.001 to 1.044, indicating no problematic multicollinearity. The Ramsey RESET and Breusch–Pagan tests suggested modest departures from linearity and homoscedasticity, respectively. However, the inclusion of nonlinear terms accounted for only an additional 1.0–2.0% of the variance. Accordingly, inferences regarding the indirect and conditional indirect effects were based on bias-corrected bootstrap confidence intervals derived from 5,000 resamples.

## 3 Results

### 3.1 Descriptive statistics and correlation analysis

Table 1 presents the descriptive statistics and bivariate correlations among the study variables. The mean scores for AI anxiety, emotion regulation, and academic motivation were 3.44 (SD = 0.801), 4.58 (SD = 0.894), and 3.13 (SD = 0.643), respectively. AI anxiety was negatively correlated with both emotion regulation (r = −0.172, *p* < 0.001) and academic motivation (r = −0.175, *p* < 0.001), whereas emotion regulation was positively correlated with academic motivation (r = 0.457, *p* < 0.001). AI contact duration showed a weak positive correlation with academic motivation (r = 0.053, *p* < 0.05), while gender was negatively correlated with academic motivation (r = −0.094, *p* < 0.001). The remaining correlations were not statistically significant.

### 3.2 Mediation effect of emotion regulation

**Figure 1:**
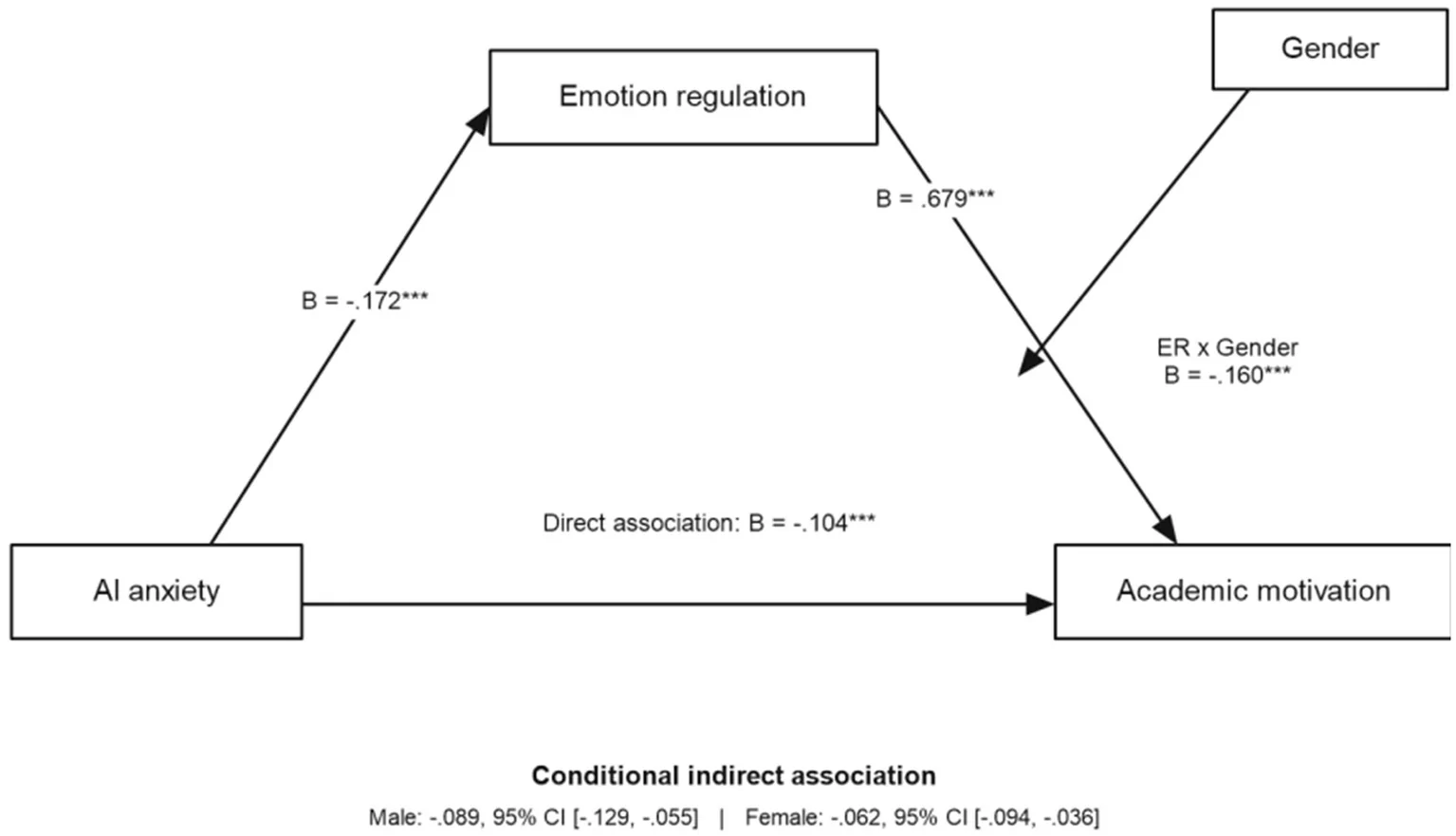
The assumed moderated mediation model.

As shown in Table 2, after controlling for daily AI use duration, AI anxiety was negatively associated with emotion regulation [*B* = −0.172, SE = 0.026, 95% CI (−0.222, −0.122), *p* < 0.001]. When AI anxiety, emotion regulation, and daily AI use duration were entered simultaneously, emotion regulation was positively associated with academic motivation [*B* = 0.439, SE = 0.023, 95% CI (0.393, 0.485), *p* < 0.001], whereas AI anxiety remained negatively associated with academic motivation [*B* = −0.101, SE = 0.023, 95% CI (−0.147, −0.056), *p* < 0.001]. The total-effect model similarly showed a significant negative association between AI anxiety and academic motivation [*B* = −0.177, SE = 0.026, 95% CI (−0.227, −0.127), *p* < 0.001]. Bootstrap analysis based on 5,000 resamples indicated a significant negative indirect association between AI anxiety and academic motivation through emotion regulation [indirect effect = −0.076, Boot SE = 0.016, 95% CI (−0.108, −0.047)]. Thus, higher AI anxiety was associated with lower emotion regulation, which, in turn, was associated with lower academic motivation. Because both the direct and indirect associations were significant, emotion regulation partially mediated the association between AI anxiety and academic motivation (Figure 1).

### 3.3 Moderating effect of gender

We used Model 14 of the PROCESS macro (Hayes, 2013) to examine whether gender moderated the indirect association between AI anxiety and academic motivation through emotion regulation. Daily AI use duration was included as a covariate.

**Figure 2:**
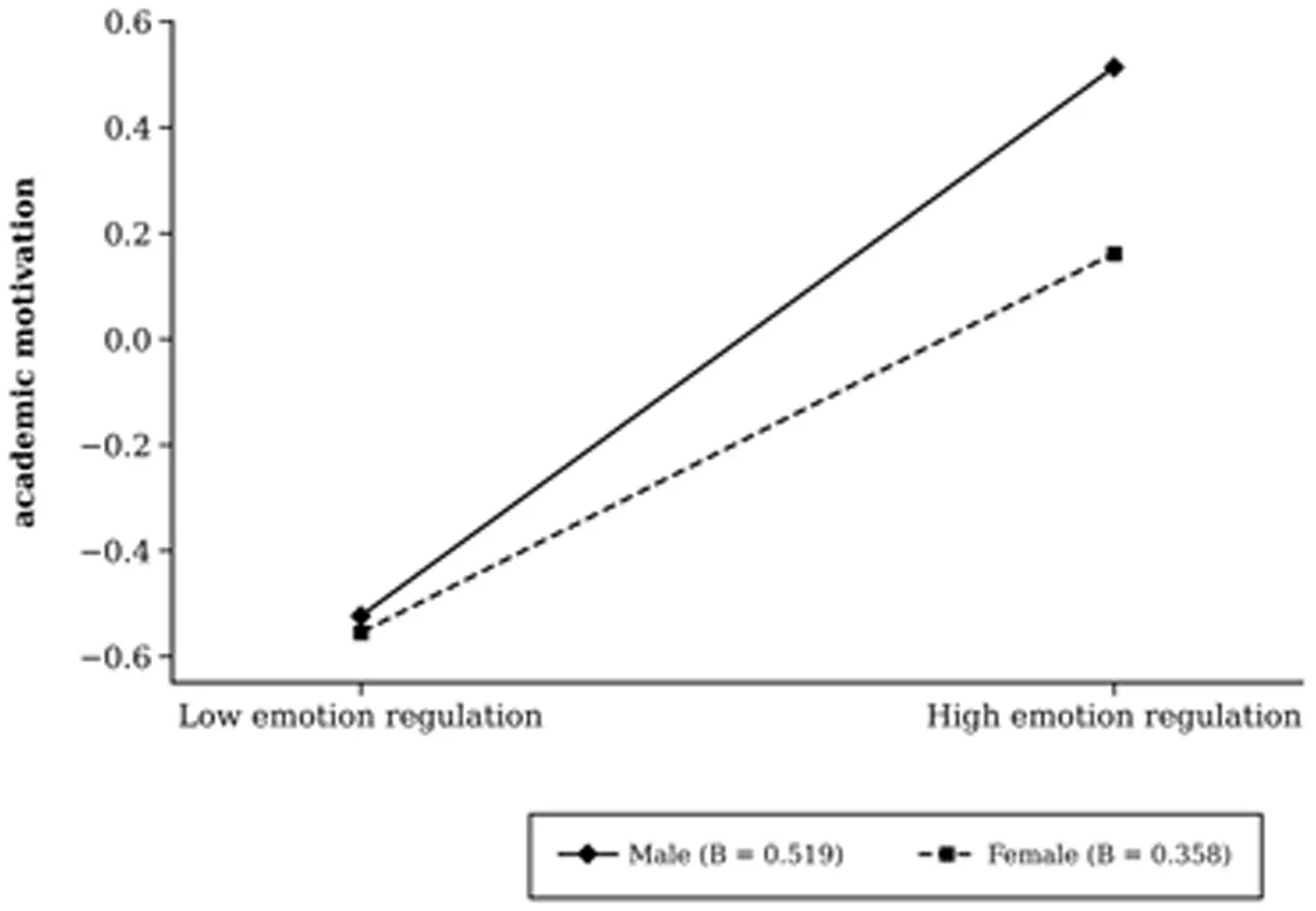
Gender moderates the direct relationship between emotion regulation and academic motivation.

As shown in Table 3, AI anxiety was negatively associated with emotion regulation (*B* = −0.172, *SE* = 0.026, *p* < 0.001). Emotion regulation, in turn, was positively associated with academic motivation (*B* = 0.679, *SE* = 0.072, *p* < 0.001). The interaction between emotion regulation and gender was significant [*B* = −0.160, *SE* = 0.046, *p* < 0.001, 95% CI (−0.250, −0.071)], indicating that gender moderated the association between emotion regulation and academic motivation. As illustrated in Figure 2, simple slope analyses showed that emotion regulation was positively associated with academic motivation among both male students (*B*simple = 0.519, *p* < 0.001) and female students (*B*simple = 0.358, *p* < 0.001). However, this positive association was stronger among male students, as indicated by the steeper slope.

Finally, bias-corrected bootstrap analyses showed that the conditional indirect association between AI anxiety and academic motivation through emotion regulation was significant for both male and female students. Specifically, the negative indirect association was stronger among male students [indirect effect = −0.089, Boot SE = 0.019, 95% CI (−0.129, −0.055)] than among female students [indirect effect = −0.062, Boot SE = 0.015, 95% CI (−0.094, −0.036)]. The index of moderated mediation was significant [index = 0.028, Boot SE = 0.012, 95% CI (0.008, 0.056)], confirming that the indirect association differed significantly by gender.

## 4 Discussion

This study examined the associations among AI anxiety, emotion regulation, academic motivation, and gender in the context of the growing integration of generative AI into higher education. AI anxiety was negatively associated with both emotion regulation and academic motivation, whereas emotion regulation was positively associated with academic motivation. A significant negative indirect association between AI anxiety and academic motivation through emotion regulation was also observed. Furthermore, gender moderated the association between emotion regulation and academic motivation, with a stronger positive association among male students. Collectively, these findings suggest that AI anxiety may constitute a psychological barrier to students’ motivational adaptation to AI-supported learning. Specifically, students with higher levels of AI anxiety may experience greater difficulty regulating their emotional responses to technological uncertainty, which may, in turn, be associated with lower academic motivation.

Daily AI use showed a weak positive correlation with academic motivation (*r* = 0.053, *p* < 0.05) and retained a small positive association in the regression model (*B* = 0.055, *p* = 0.026). This finding should be interpreted cautiously, as most participants reported using AI for less than one hour per day, resulting in a restricted and uneven distribution of usage duration. The observed association may therefore partly reflect the usage characteristics of the present sample and should not be interpreted as evidence that greater AI use necessarily promotes academic motivation. Moreover, usage duration alone does not capture the purpose, quality, or academic relevance of students’ AI engagement. Future research should distinguish between academic and non-academic AI use and examine how the frequency, purpose, and quality of AI engagement are differentially associated with academic motivation, because ChatGPT engagement may depend on perceived usefulness, task design and learning context rather than exposure duration alone (Gao et al., 2024; Jin and Sercu, 2025).

### 4.1 Complexity of the relationship between AI anxiety and academic motivation

This study found that AI anxiety was negatively associated with academic motivation, supporting H1. The zero-order correlation was negative, and AI anxiety retained a significant negative association with academic motivation after emotion regulation and daily AI use duration were included in the model. These findings suggest that AI anxiety may represent a psychological barrier to students’ motivational adaptation to AI-supported learning.

From a self-regulated learning perspective, persistent concerns about technological reliability, academic integrity, competence displacement, and future development may consume the cognitive and emotional resources required for effective learning regulation. Students with higher AI anxiety may experience lower perceived control over AI-supported learning and greater uncertainty about their ability to meet changing academic demands. Such experiences may interfere with goal setting, effort regulation, and sustained willingness to learn. The findings therefore support an interpretation of AI anxiety as a potentially resource-depleting experience rather than a motivational stress signal (Galindo-Domínguez et al., 2026; Silvola et al., 2025).

Nevertheless, the negative association should not be interpreted as evidence that all concerns about AI are detrimental. A certain degree of awareness of AI-related risks may encourage responsible use and critical reflection. However, the present data do not support the proposed “stress-mobilisation” interpretation because AI anxiety was negatively associated with academic motivation in both the correlation and regression analyses. Future research should examine whether the association is nonlinear or varies according to AI literacy, self-efficacy, perceived value, technological trust, and social support (Cao et al., 2026; Chen C. et al., 2025; Tarhini et al., 2026; Uludağ et al., 2025; Yang, 2026; Zahid et al., 2026).

### 4.2 Mediating role of emotion regulation

Emotion regulation significantly mediated the association between AI anxiety and academic motivation, supporting H2. Specifically, AI anxiety was negatively associated with emotion regulation, whereas emotion regulation was positively associated with academic motivation. The significant negative indirect association indicates that higher AI anxiety was associated with lower emotion regulation, which, in turn, was associated with lower academic motivation. Because the direct association between AI anxiety and academic motivation remained significant after emotion regulation was included, the results were consistent with partial mediation.

First, the negative association between AI anxiety and emotion regulation supported H2a. Students experiencing greater uncertainty regarding AI-generated content, academic-use boundaries, competence comparison, or occupational displacement may have greater difficulty managing tension and worry. Persistent AI-related concerns may consume attentional resources, reduce perceived emotional control, and constrain the effective use of adaptive regulation strategies. Thus, rather than activating stronger emotion regulation, higher AI anxiety was associated with reduced emotion-regulation capacity in the present sample.

Second, emotion regulation was positively associated with academic motivation, supporting H2b. Students with stronger emotion-regulation capacities may be better able to reduce emotional interference, maintain perceived control over learning, and sustain goal-directed effort when confronting AI-related uncertainty. By contrast, difficulties in regulating anxiety and frustration may increase cognitive load and weaken motivational persistence. This interpretation is consistent with previous studies linking emotion regulation to academic motivation, approaches to learning, academic performance, and lower academic burnout (Garn and Stenling, 2024; Liang and Mao, 2025; Rentzios et al., 2025; Romo et al., 2025; Wang et al., 2025).

Finally, the significant indirect association suggests that emotion regulation constitutes one psychological pathway through which AI anxiety is related to academic motivation. The results do not indicate that emotion regulation transforms AI anxiety into a motivational resource. Instead, they suggest a resource-depletion process: higher AI anxiety is associated with poorer emotion regulation, which is subsequently associated with lower academic motivation. This finding extends research on AI-supported learning by highlighting that students’ adaptation depends not only on their ability to use AI tools but also on their capacity to manage the uncertainty and emotional demands accompanying such use.

From a study demands–resources perspective, AI-supported learning provides new instructional resources while simultaneously introducing demands related to information evaluation, academic integrity, self-monitoring, and technological adaptation. Emotion regulation may function as a personal resource that helps students maintain psychological stability and goal orientation under these demands. Accordingly, AI literacy initiatives should extend beyond technical skills to include support for emotional adaptation, stress management, critical evaluation, and learning-goal regulation (Chen et al., 2026; Guan et al., 2025; Lu et al., 2026; Luo and Yusuf, 2026; Mirriahi et al., 2025; Shi et al., 2025).

### 4.3 Moderating role of gender

Consistent with H3, gender significantly moderated the association between emotion regulation and academic motivation. Emotion regulation was positively associated with academic motivation among both male (B = 0.519, *p* < 0.001) and female students (B = 0.358, *p* < 0.001), but the association was significantly stronger among male students. Thus, emotion regulation was important for academic motivation in both groups, but it appeared to represent a relatively stronger motivational resource for male students in this sample. Related research indicates that students may differ in their perceptions of AI-related risks and technological trust (Arthur et al., 2024). Gender differences have also been observed in intentions and patterns of AI use (Strzelecki and ElArabawy, 2024).

Gender-role socialisation provides one possible explanation for this difference. Male students may be socialised to emphasise emotional control, independence, goal persistence and problem-focused coping when facing academic or technological challenges. Consequently, their ability to regulate negative emotions may be more directly associated with maintaining learning goals, perceived control and academic motivation. By contrast, female students may regulate academic stress through a broader combination of intrapersonal and interpersonal resources, including emotional expression, teacher feedback, peer communication and social support. The availability of these additional resources may reduce the relative strength of the association between individual emotion regulation and academic motivation. This explanation extends the findings by suggesting that gender may function as a boundary condition that shapes how emotional resources are translated into motivational outcomes.

These findings highlight the value of combining emotion-regulation support with teacher guidance, peer support and clear expectations regarding AI use. However, the stronger association among male students should not be interpreted as evidence that male students have better emotion-regulation abilities or that emotion regulation is unimportant for female students. The result concerns a difference in the strength of the relationship, rather than a difference in mean levels or abilities. Because coping strategies and social support were not directly measured, the proposed explanation remains tentative. Given the binary measurement of gender and the cross-sectional design, future research should examine whether social support, AI self-efficacy and coping strategies account for this gender difference (Tarhini et al., 2026; Zahid et al., 2026).

### 4.4 Theoretical contributions

This study makes three theoretical contributions. First, the findings support a central proposition of self-regulated learning (SRL) theory—that emotional and motivational regulation are interdependent components of students’ adaptation to changing learning demands (Boekaerts, 1999; Efklides, 2011; Fong et al., 2024; Theobald, 2021; Zimmerman, 2000). The negative indirect association between AI anxiety and academic motivation through emotion regulation suggests that difficulty managing AI-related emotional demands may constrain students’ motivational regulation.

Second, this study extends SRL theory to AI-supported learning by conceptualising AI anxiety as a contextual demand rather than treating AI solely as a learning resource. The findings indicate that effective human–AI collaboration depends not only on technical competence but also on students’ capacity to regulate emotional and metacognitive processes. This interpretation complements evidence that productive AI-supported learning requires the integration of self-regulation and AI literacy (Galindo-Domínguez et al., 2026; Yang, 2026; Zhang and Chen, 2026).

Third, the moderating role of gender refines the assumption that self-regulatory processes operate uniformly across learners. Although emotion regulation was positively associated with academic motivation in both groups, the association was stronger among male students. SRL theory may therefore benefit from greater attention to individual and contextual differences in how emotional resources are mobilised. Given the cross-sectional design, however, these findings extend and refine rather than revise the core propositions of SRL theory.

### 4.5 Educational implications

The findings suggest that AI literacy education may benefit from extending beyond technical proficiency to include metacognitive and psychological competencies. Universities could consider supporting students in planning, monitoring, and evaluating their use of generative AI, critically verifying AI-generated information, revising ineffective prompts, and reflecting on how AI affects their learning decisions. Such instruction may be associated with more strategic engagement with AI and less uncritical dependence on automated outputs (Chen et al., 2026; EL Hosayny et al., 2026; Lu and Zeng, 2026; Lu et al., 2026; Luo and Yusuf, 2026; Zhang and Chen, 2026). Emotion-regulation support could also be considered within AI-related courses as a possible means of helping students manage technological uncertainty, maintain perceived control, and sustain academic motivation. This suggestion is consistent with evidence linking AI-supported learning to self-regulation, resilience, and learner autonomy (Zhang, 2025).

AI literacy education may also need to address responsible AI use and academic integrity explicitly. Universities could provide clear guidance regarding acceptable AI assistance, authorship, disclosure, source verification, and the ethical boundaries of AI-assisted work. Process-oriented assessment, reflective AI-use statements, and structured dialogue between students and instructors may help students use AI as a learning aid without relinquishing intellectual responsibility (Chkarka and Fatmi, 2026; Gonsalves, 2025; Winstone et al., 2026; Zhang et al., 2024). Institutions could further consider combining emotion-regulation support with teacher guidance, peer support, and differentiated learning assistance, while avoiding assumptions that all students experience or regulate AI anxiety in the same way.

## 5 Limitations and future research

This study has several limitations. First, its cross-sectional design does not permit causal conclusions or establish the temporal ordering of AI anxiety, emotion regulation and academic motivation. Reverse relationships are also possible. Future studies should employ longitudinal, cross-lagged or experimental designs to examine the dynamic relationships among these variables.

Second, the study relied on self-report measures and used composite scores in the PROCESS analysis. Although CFA supported the measurement structure of the scales, PROCESS treats variables as observed scores and does not explicitly account for measurement error. The findings may also be affected by common method bias, social desirability and subjective reporting. Future studies should use latent-variable structural equation modelling and incorporate AI interaction logs and learning-platform records (Guan et al., 2025). Interviews and academic outcomes could also be included to provide multi-method validation (Mirriahi et al., 2025). Process-based data may further reveal how students regulate anxiety and adjust their AI-use strategies during specific learning tasks (Tao et al., 2026).

Third, the three instruments were administered in a fixed order—emotion regulation, academic motivation and AI anxiety—rather than in a randomized order. Therefore, potential order, priming and fatigue effects cannot be excluded. Future studies should randomize or counterbalance the order of the instruments.

Fourth, AI anxiety was treated as a unidimensional construct. However, recent research suggests that AI anxiety may involve learning-related anxiety, occupational-replacement concerns and sociotechnical uncertainty (Wang et al., 2024). Ethical concerns may also constitute a distinct source of anxiety about AI use (Zhao et al., 2025). Future research should examine whether these dimensions are differently associated with deep motivation, surface motivation and amotivation (Cao et al., 2026).

Fifth, participants’ pre-existing AI literacy was not measured or controlled. AI literacy may be associated with both lower AI anxiety and stronger academic motivation, and thus may represent a potential confounding variable. Future studies should assess AI literacy and examine its role as a covariate, moderator or explanatory mechanism.

Finally, the psychological mechanisms underlying the moderating role of gender require further investigation. Future studies could examine AI self-efficacy, social support, academic discipline and learning context as potential explanatory factors. Institutional context and social support may shape students’ adaptation to generative AI (Budhathoki et al., 2024). Teacher modelling and self-regulated learning may also influence students’ AI-related experiences (Shi et al., 2025). Because the sample was limited to Chinese university students, multi-country and multi-institutional studies are needed to assess the generalisability of the findings (Strzelecki and ElArabawy, 2024).

## Data Availability

The original contributions presented in the study are included in the article/Supplementary material, further inquiries can be directed to the corresponding author.

## References

[ref1] Aldao A. Nolen-Hoeksema S. Schweizer S. (2010). Emotion-regulation strategies across psychopathology: a meta-analytic review. Clin. Psychol. Rev. 30, 217–237. doi: 10.1016/j.cpr.2009.11.004, 20015584

[ref2] Al-Mamary Y. H. Abubakar A. A. (2025). Empowering ChatGPT adoption in higher education: a comprehensive analysis of university students' intention to adopt artificial intelligence using self-determination and technology-to-performance chain theories. Internet High. Educ. 66:101015. doi: 10.1016/j.iheduc.2025.101015

[ref3] Alshaibani M. H. Al-Rahmi W. M. Tawafak R. M. (2025). Psychometric validation of the artificial intelligence anxiety scale: a confirmatory factor analysis for academic research. Contemp. Educ. Technol. 17:ep595. doi: 10.30935/cedtech/17309

[ref4] Arthur F. Salifu I. Abam Nortey S. (2024). Predictors of higher education students’ behavioural intention and usage of ChatGPT: the moderating roles of age, gender and experience. Interact. Learn. Environ. 1–27, 1–27. doi: 10.1080/10494820.2024.2362805, 37339054

[ref5] Bakker A. B. (2024). Study demands–resources theory: understanding student well-being in higher education. Educ. Psychol. Rev. 36:92. doi: 10.1007/s10648-024-09940-8

[ref6] Bakker A. B. Demerouti E. (2017). Job demands–resources theory: taking stock and looking forward. J. Occup. Health Psychol. 22, 273–285. doi: 10.1037/ocp0000056, 27732008

[ref7] Ben-Eliyahu A. Linnenbrink-Garcia L. (2013). Extending self-regulated learning to include self-regulated emotion strategies. Motiv. Emot. 37, 558–573. doi: 10.1007/s11031-012-9332-3

[ref8] Biggs J. Kember D. Leung D. Y. P. (2001). The revised two-factor study process questionnaire: R-SPQ-2F. Br. J. Educ. Psychol. 71, 133–149. doi: 10.1348/000709901158433, 11307705

[ref9] Bittle K. El-Gayar O. (2025). Generative AI and academic integrity in higher education: a systematic review and research agenda. Information 16:296. doi: 10.3390/info16040296, 30654563

[ref10] Boekaerts M. (1999). Self-regulated learning: where we are today. Int. J. Educ. Res. 31, 445–457. doi: 10.1016/S0883-0355(99)00014-2

[ref11] Budhathoki T. Zirar A. Njoya E. T. Timsina A. (2024). ChatGPT adoption and anxiety: a cross-country analysis utilising the unified theory of acceptance and use of technology (UTAUT). Stud. High. Educ. 49, 831–846. doi: 10.1080/03075079.2024.2333937

[ref12] Bureau J. S. Howard J. L. Chong J. X. Y. Guay F. (2022). Pathways to student motivation: a meta-analysis of antecedents of autonomous and controlled motivations. Rev. Educ. Res. 92, 46–72. doi: 10.3102/00346543211042426, 35330866 PMC8935530

[ref13] Cao K. Wang P. Jiang X. (2026). AI anxiety and adoption intention in higher education based on an extended TAM-UTAUT and PLS-SEM analysis. Sci. Rep. 16:3672. doi: 10.1038/s41598-026-35823-9 PMC12881405 41545432

[ref14] Chan C. K. Y. Hu W. (2023). Students’ voices on generative AI: perceptions, benefits, and challenges in higher education. Int. J. Educ. Technol. High. Educ. 20:43. doi: 10.1186/s41239-023-00411-8

[ref15] Chen S. Cheung A. C. K. (2025). Effect of generative artificial intelligence on university students learning outcomes: a systematic review and meta-analysis. Educ. Res. Rev. 49:100737. doi: 10.1016/j.edurev.2025.100737

[ref16] Chen J. He M. Sun J. (2025). AI anxiety and knowledge payment: the roles of perceived value and self-efficacy. BMC Psychol. 13:208. doi: 10.1186/s40359-025-02510-9, 40050942 PMC11887247

[ref17] Chen C. Hu W. Wei X. (2025). From anxiety to action: exploring the impact of artificial intelligence anxiety and artificial intelligence self-efficacy on motivated learning of undergraduate students. Interact. Learn. Environ. 33, 3162–3177. doi: 10.1080/10494820.2024.2440877

[ref18] Chen S. Zhang L. Li A. W. (2026). Leveraging self-regulated learning to support university students’ feedback literacy in blended learning environments. Assess. Eval. High. Educ. 51, 174–193. doi: 10.1080/02602938.2025.2546349

[ref19] Chkarka F. Fatmi H. (2026). Comparative examination of master’s students’ and faculty members’ maintenance of academic integrity in the age of AI. J Academic Ethics 24:10. doi: 10.1007/s10805-025-09684-2, 30311153

[ref20] Crompton H. Burke D. (2023). Artificial intelligence in higher education: the state of the field. Int. J. Educ. Technol. High. Educ. 20:22. doi: 10.1186/s41239-023-00392-8

[ref21] Eccles J. S. Wigfield A. (2020). From expectancy-value theory to situated expectancy-value theory: a developmental, social cognitive, and sociocultural perspective on motivation. Contemp. Educ. Psychol. 61:101859. doi: 10.1016/j.cedpsych.2020.101859, 38826717

[ref22] Efklides A. (2011). Interactions of metacognition with motivation and affect in self-regulated learning: the MASRL model. Educ. Psychol. 46, 6–25. doi: 10.1080/00461520.2011.538645

[ref23] EL Hosayny O. Razkane H. Yeou M. (2026). Metacognitive regulation and ChatGPT-assisted writing: introducing an interactive model for cultivating strategic engagement. Discov. Educ. 5:246. doi: 10.1007/s44217-026-01282-7

[ref24] Fong C. J. Altan S. Gonzales C. Kirmizi M. Adelugba S. F. Kim Y. E. (2024). Stay motivated and carry on: a meta-analytic investigation of motivational regulation strategies and academic achievement, motivation, and self-regulation correlates. J. Educ. Psychol. 116, 997–1018. doi: 10.1037/edu0000886

[ref25] Galindo-Domínguez H. Delgado N. Sainz-de-la-Maza M. Etxabe J. M. (2026). Self-regulation and overreliance on artificial intelligence: unpacking a paradox through a mixed-methods study in higher education. Comput. Hum. Behav. 181:108985. doi: 10.1016/j.chb.2026.108985

[ref26] Gao L. López-Pérez M. E. Melero-Polo I. Trifu A. (2024). Ask ChatGPT first! Transforming learning experiences in the age of artificial intelligence. Stud. High. Educ. 49, 2772–2796. doi: 10.1080/03075079.2024.2323571

[ref27] Garn A. C. Stenling A. (2024). University students’ daily motivation regulation: within- and between-level relations to academic functioning. Educ. Psychol. 44, 227–246. doi: 10.1080/01443410.2024.2331754

[ref28] Gonsalves C. (2025). Addressing student non-compliance in AI use declarations: implications for academic integrity and assessment in higher education. Assess. Eval. High. Educ. 50, 592–606. doi: 10.1080/02602938.2024.2415654

[ref29] Gross J. J. (1998). The emerging field of emotion regulation: an integrative review. Rev. Gen. Psychol. 2, 271–299. doi: 10.1037/1089-2680.2.3.271

[ref30] Gross J. J. (2015). Emotion regulation: current status and future prospects. Psychol. Inq. 26, 1–26. doi: 10.1080/1047840X.2014.940781

[ref31] Gross J. J. John O. P. (2003). Individual differences in two emotion regulation processes: implications for affect, relationships, and well-being. J. Pers. Soc. Psychol. 85, 348–362. doi: 10.1037/0022-3514.85.2.348, 12916575

[ref32] Guan R. Raković M. Chen G. Gašević D. (2025). How educational chatbots support self-regulated learning? A systematic review of the literature. Educ. Inf. Technol. 30, 4493–4518. doi: 10.1007/s10639-024-12881-y

[ref33] Guay F. (2022). Applying self-determination theory to education: regulation types, psychological needs, and autonomy-supporting behaviors. Can. J. Sch. Psychol. 37, 75–92. doi: 10.1177/08295735211055355

[ref34] Güner Ö. Aydın M. S. (2026). Artificial intelligence anxiety among nursing and midwifery students: the role of resistance to change—a cross-sectional study. BMC Med. Educ. 26. doi: 10.1186/s12909-026-08754-2, 41749216 PMC13041113

[ref35] Harley J. M. Pekrun R. Taxer J. L. Gross J. J. (2019). Emotion regulation in achievement situations: an integrated model. Educ. Psychol. 54, 106–124. doi: 10.1080/00461520.2019.1587297

[ref36] Hayes A. F. (2013). Introduction to Mediation, Moderation, and Conditional process Analysis: A Regression-based Approach. New York, NY: The Guilford Press.

[ref37] Hmoud M. Swaity H. Hamad N. Karram O. Daher W. (2024). Higher education students’ task motivation in the generative artificial intelligence context: the case of ChatGPT. Information 15:33. doi: 10.3390/info15010033, 30654563

[ref38] Howard J. L. Bureau J. S. Guay F. Chong J. X. Y. Ryan R. M. (2021). Student motivation and associated outcomes: a meta-analysis from self-determination theory. Perspect. Psychol. Sci. 16, 1300–1323. doi: 10.1177/1745691620966789, 33593153

[ref39] Hu W. W. (2026). University student perspectives on generative AI: reconfiguring competence, fairness, and authorship in academic work. Stud. High. Educ. 1–19, 1–19. doi: 10.1080/03075079.2026.2658738, 37339054

[ref40] Iuga I. A. David O. A. (2024). Emotion regulation and academic burnout among youth: a meta-analysis. Educ. Psychol. Rev. 36:99. doi: 10.1007/s10648-024-09930-w

[ref42] Jin Y. Sercu L. (2025). ChatGPT interventions in higher education: a systematic review of experimental studies. J. Comput. Assist. Learn. 41:e70072. doi: 10.1111/jcal.70072

[ref43] Karaaslan F. Erişken Y. (2026). Artificial intelligence related anxiety among dental students: associations with demographics and AI use behaviors. BMC Med. Educ. 26:969. doi: 10.1186/s12909-026-09161-3 PMC13267659 42050484

[ref44] Kasneci E. Sessler K. Küchemann S. Bannert M. Dementieva D. Fischer F. . (2023). ChatGPT for good? On opportunities and challenges of large language models for education. Learn. Individ. Differ. 103:102274. doi: 10.1016/j.lindif.2023.102274

[ref45] Kim J. Klopfer M. Grohs J. R. Eldardiry H. Weichert J. Cox L. A. II . (2025). Examining faculty and student perceptions of generative AI in university courses. Innov. High. Educ. 50, 1281–1313. doi: 10.1007/s10755-024-09774-w

[ref46] Kofinas A. K. Tsay C. H. Pike D. (2025). The impact of generative AI on academic integrity of authentic assessments within a higher education context. Br. J. Educ. Technol. 56, 2522–2549. doi: 10.1111/bjet.13585

[ref47] Kryshko O. Fleischer J. Grunschel C. Leutner D. (2023). University students’ self-efficacy for motivational regulation, use of motivational regulation strategies, and satisfaction with academic studies: exploring between-person and within-person associations. J. Educ. Psychol. 115, 571–588. doi: 10.1037/edu0000785

[ref48] Liang H. Mao X. (2025). Emotion regulation and perceptions of academic stress as key predictors of academic motivation in second language learning. PLoS One 20:e0327071. doi: 10.1371/journal.pone.0327071, 40824942 PMC12360526

[ref49] Litalien D. Tóth-Király I. Guay F. Morin A. J. S. (2024). PhD students’ motivation profiles: a self-determination theory perspective. Contemp. Educ. Psychol. 77:102279. doi: 10.1016/j.cedpsych.2024.102279

[ref50] Lu Q. Yao Y. Xiao L. Zhu X. Yin H. (2026). Can GenAI help undergraduate students become independent writers-an intervention study on its effects on writing motivation, feedback literacy, and self-regulated learning strategies. Educ. Psychol. 1–27, 1–27. doi: 10.1080/01443410.2026.2654526, 37339054

[ref51] Lu D. Zeng Y. (2026). Leveraging self-regulated learning to develop L2 students’ feedback literacy in GenAI-mediated writing environments: an intervention study. Assess. Eval. High. Educ. 51, 1–19. doi: 10.1080/02602938.2026.2653887

[ref52] Lund B. D. Lee T. H. Mannuru N. R. Arutla N. R. (2025). AI and academic integrity: exploring student perceptions and implications for higher education. J. Acad. Ethics 23, 1545–1565. doi: 10.1007/s10805-025-09604-5

[ref53] Luo X. Wang S. Hew K. F. (2026). Development and implementation of a generative AI-based personalized recommender system to improve students’ self-regulated learning and academic performance. Comput. Educ. 241:105486. doi: 10.1016/j.compedu.2025.105486

[ref54] Luo L. Yusuf A. (2026). Bridging AI and pedagogy: how AI-adaptive feedback shapes Chinese EFL students’ writing engagement, metacognitive writing strategies, and writing performance. Assess. Eval. High. Educ. 51, 508–531. doi: 10.1080/02602938.2025.2548919

[ref55] Marques H. Brites R. Nunes O. Hipólito J. Brandão T. (2023). Attachment, emotion regulation, and burnout among university students: a mediational hypothesis. Educ. Psychol. 43, 344–362. doi: 10.1080/01443410.2023.2212889

[ref56] McRae K. Ochsner K. N. Mauss I. B. Gabrieli J. J. D. Gross J. J. (2008). Gender differences in emotion regulation: an fMRI study of cognitive reappraisal. Group Process. Intergroup Relat. 11, 143–162. doi: 10.1177/1368430207088035, 29743808 PMC5937254

[ref57] Mega C. Ronconi L. De Beni R. (2014). What makes a good student? How emotions, self-regulated learning, and motivation contribute to academic achievement. J. Educ. Psychol. 106, 121–131. doi: 10.1037/a0033546

[ref58] Mirriahi N. Marrone R. Barthakur A. Gabriel F. Colton J. Yeung T. N. . (2025). The relationship between students’ self-regulated learning skills and technology acceptance of GenAI. Australas. J. Educ. Technol. 41, 16–33. doi: 10.14742/ajet.10006

[ref59] Moorhouse B. L. Wan Y. Wu C. Wu M. Ho T. Y. (2025). Generative AI tools and empowerment in L2 academic writing. System 133:103779. doi: 10.1016/j.system.2025.103779, 38826717

[ref60] O’Dea X. (2024). Generative AI: is it a paradigm shift for higher education. Stud. High. Educ. 49, 811–816. doi: 10.1080/03075079.2024.2332944

[ref61] Pallant J. L. Blijlevens J. Campbell A. Jopp R. (2025). Mastering knowledge: the impact of generative AI on student learning outcomes. Stud. High. Educ. 51, 1–22. doi: 10.1080/03075079.2025.2487570

[ref63] Pekrun R. (2006). The control-value theory of achievement emotions: assumptions, corollaries, and implications for educational research and practice. Educ. Psychol. Rev. 18, 315–341. doi: 10.1007/s10648-006-9029-9

[ref64] Pekrun R. Goetz T. Titz W. Perry R. P. (2002). Academic emotions in students’ self-regulated learning and achievement: a program of qualitative and quantitative research. Educ. Psychol. 37, 91–105. doi: 10.1207/S15326985EP3702_4, 42502907

[ref65] Rentzios C. Karagiannopoulou E. Ntritsos G. (2025). Academic emotions, emotion regulation, academic motivation, and approaches to learning: a person-centered approach. Behavioral Sciences 15:900. doi: 10.3390/bs15070900, 40723684 PMC12292873

[ref66] Romo J. Pérez J. C. Cumsille P. Hollenstein T. Olaya-Torres A. Rodríguez-Rivas M. E. . (2025). Emotion regulation strategies and academic achievement among secondary and university students: a systematic review and meta-analysis. Educ. Psychol. Rev. 37:80. doi: 10.1007/s10648-025-10054-y, 30311153

[ref67] Schweder S. Raufelder D. (2022). Students’ interest and self-efficacy and the impact of changing learning environments. Contemp. Educ. Psychol. 70:102082. doi: 10.1016/j.cedpsych.2022.102082

[ref68] Schwinger M. Trautner M. Pütz N. Fabianek S. Lemmer G. Lauermann F. . (2022). Why do students use strategies that hurt their chances of academic success-a meta-analysis of antecedents of academic self-handicapping. J. Educ. Psychol. 114, 576–596. doi: 10.1037/edu0000706

[ref69] Shi J. Liu W. Hu K. (2025). Exploring how AI literacy and self-regulated learning relate to student writing performance and well-being in generative AI-supported higher education. Behavioral Sciences 15:705. doi: 10.3390/bs15050705, 40426482 PMC12108985

[ref70] Silvola A. Kajamaa A. Merikko J. Muukkonen H. (2025). AI-mediated sensemaking in higher education students’ learning processes: tensions, sensemaking practices, and AI-assigned purposes. Br. J. Educ. Technol. 56, 2001–2018. doi: 10.1111/bjet.13606

[ref71] Strzelecki A. (2024). To use or not to use ChatGPT in higher education-a study of students’ acceptance and use of technology. Interact. Learn. Environ. 32, 5142–5155. doi: 10.1080/10494820.2023.2209881

[ref72] Strzelecki A. ElArabawy S. (2024). Investigation of the moderation effect of gender and study level on the acceptance and use of generative AI by higher education students: comparative evidence from Poland and Egypt. Br. J. Educ. Technol. 55, 1209–1230. doi: 10.1111/bjet.13425

[ref73] Tamres L. K. Janicki D. Helgeson V. S. (2002). Sex differences in coping behavior: a meta-analytic review and an examination of relative coping. Personal. Soc. Psychol. Rev. 6, 2–30. doi: 10.1207/S15327957PSPR0601_1

[ref74] Tao L. Song Y. Fu J. (2026). Exploring students' self-regulated learning behavioural patterns and perceptions in an English speaking task within a generative AI-supported immersive VR environment. Comput. Educ. 242:105515. doi: 10.1016/j.compedu.2025.105515

[ref75] Tarhini A. Al-Sharafi M. A. Al Lawati H. (2026). Role of AI characteristics, AI literacy, and institutional policy in shaping generative AI use and learning outcomes among higher education students. Interact. Learn. Environ. 1–26, 1–26. doi: 10.1080/10494820.2026.2628249, 37339054

[ref76] Teke B. (2026). Can artificial intelligence anxiety serve as a facilitator: the relationship between pre-service teachers’ AI anxiety, their motivated learning and AI self-efficacy. Interact. Learn. Environ. 34, 3086–3100. doi: 10.1080/10494820.2025.2559930

[ref77] Theobald M. (2021). Self-regulated learning training programs enhance university students’ academic performance, self-regulated learning strategies, and motivation: a meta-analysis. Contemp. Educ. Psychol. 66:101976. doi: 10.1016/j.cedpsych.2021.101976

[ref78] Uludağ F. Kılıç E. Çelik H. E. (2025). Artificial intelligence, social influence, and AI anxiety: analyzing the intentions of science doctoral students to use ChatGPT with PLS-SEM. Hum. Soc. Sci. Commun. 12:1308. doi: 10.1057/s41599-025-05641-x

[ref79] Venkatesh V. Morris M. G. Davis G. B. Davis F. D. (2003). User acceptance of information technology: toward a unified view. MIS Q. 27, 425–478. doi: 10.2307/30036540

[ref80] Wang L. Shi Y. Yang X. Chen Y. Chen Q. Yang Y. . (2025). Achievement goal profiles and anxiety among university students: the indirect roles of emotion regulation strategies. Educ. Psychol. 45, 641–660. doi: 10.1080/01443410.2025.2499568

[ref82] Wang Y.-M. Wei C.-L. Lin H.-H. Wang S.-C. Wang Y.-S. (2024). What drives students’ AI learning behavior: a perspective of AI anxiety. Interact. Learn. Environ. 32, 1–17. doi: 10.1080/10494820.2022.2153147, 37339054

[ref83] Webb T. L. Miles E. Sheeran P. (2012). Dealing with feeling: a meta-analysis of the effectiveness of strategies derived from the process model of emotion regulation. Psychol. Bull. 138, 775–808. doi: 10.1037/a0027600, 22582737

[ref84] Winstone N. E. Gravett K. Elkington S. (2026). Black box assessment: rethinking integrity and learning for a time of generative AI. Assess. Eval. High. Educ. 1–18, 1–18. doi: 10.1080/02602938.2026.2661365, 37339054

[ref85] Yang S. C. (2026). Integrating technology acceptance, self-determination, and self-regulation: a structural model of generative AI-supported learning and competence. Comput. Hum. Behav. 179:108933. doi: 10.1016/j.chb.2026.108933

[ref86] Zahid H. Danish M. Ali S. Huo C. Khalid N. (2026). Generative AI chatbots in higher education: addiction, cognitive offloading, and the role of AI literacy. Interact. Learn. Environ. 1–18, 1–18. doi: 10.1080/10494820.2026.2677727, 37339054

[ref87] Zeijen M. E. L. Brenninkmeijer V. Peeters M. C. W. Mastenbroek N. J. J. M. (2024). The role of personal demands and personal resources in enhancing study engagement and preventing study burnout. Span. J. Psychol. 27:Article e5. doi: 10.1017/SJP.2024.5 38454632

[ref88] Zhang Z. (2025). The role of artificial intelligence tools on Chinese EFL learners’ self-regulation, resilience and autonomy. Eur. J. Educ. 60:Article e70127. doi: 10.1111/ejed.70127

[ref89] Zhang L. Amos C. Pentina I. (2024). Interplay of rationality and morality in using ChatGPT for academic misconduct. Behav. Inf. Technol. Advance online publication 44, 491–507. doi: 10.1080/0144929X.2024.2325023

[ref90] Zhang L. Chen S. C. (2026). Towards human–AI collaborative learning: synergizing self-regulation and artificial-intelligence literacy. Educ. Inf. Technol. 31, 1879–1907. doi: 10.1007/s10639-025-13880-3

[ref91] Zhang B. Li W. Jou M. (2026). The influence of generative artificial intelligence support on learning anxiety and academic expectation stress: the mediating role of self-efficacy. Interact. Learn. Environ. 34, 3557–3573. doi: 10.1080/10494820.2025.2570493

[ref92] Zhao L. Xu Y. Zhou S. K. (2025). “Positive” or “threatened”-the impact of the features in generative artificial intelligence on continued behavior. Comput. Hum. Behav. 168:108654. doi: 10.1016/j.chb.2025.108654

[ref93] Zimmerman B. J. (2000). “Attaining self-regulation: a social cognitive perspective,” in Handbook of Self-Regulation, eds. Boekaerts M. Pintrich P. R. Zeidner M. (San Diego, CA: Academic Press), 13–39. doi: 10.1016/B978-012109890-2/50031-7

