# Supplementary material for: The relationship between AI anxiety and academic motivation among university students: the mediating role of emotion regulation and the moderating role of gender
Source: Front Psychol. 2026 Sep 4;17:1918525. doi: 10.3389/fpsyg.2026.1918525 (PMC13585744; doi:10.3389/fpsyg.2026.1918525)
Supplement: Supplementary file 1 [file Data_Sheet_1.pdf]

lementary Table S1：Diagnostic statistics for the regression models

Panel A. Distributional characteristics of the continuous variables

**Variable**	**Skewness**	**Kurtosis**	**Shapiro–Wilk *W***	***p***
AI anxiety	−0.148	0.319	0.962	< .001
Emotion regulation	−0.914	2.124	0.935	< .001
Academic motivation	−0.022	1.94	0.946	< .001

Panel B. Linearity and homoscedasticity diagnostics

**Regression model**	**RESET *F***	**Δ*R*²**	**BP χ²**	***df***	***p***
Emotion regulation model	7.666***	0.01	68.524	2	< .001
Mediation outcome model	19.790***	0.02	49.846	3	< .001
Moderated mediation outcome model	17.614***	0.018	55.043	5	< .001

Panel C. Multicollinearity diagnostic

Model	Predictor	VIF	Tolerance
Mediation model	AI anxiety	1.031	0.97
	Emotion regulation	1.031	0.97
	AI contact duration	1.001	0.999
Moderated mediation model	AI anxiety	1.033	0.968
	Emotion regulation	1.044	0.958
	Gender	1.001	0.999
	Emotion regulation * Gender	1.017	0.983
	AI contact duration	1.007	0.993

Note. N = 1,484. RESET = Ramsey Regression Equation Specification Error Test;

BP = Breusch–Pagan test; VIF = variance inflation factor. Gender and AI contact duration were not assessed for normality because they were categorical or ordinal variables. ΔR^2 represents the additional variance explained by the nonlinear terms in the RESET test. [1]The moderator was centered before constructing the interaction term.

*** p < .001.
